# Aripiprazole induced reversible ptosis anda oromandibular dystonia

**DOI:** 10.1192/j.eurpsy.2024.1454

**Published:** 2024-08-27

**Authors:** S. E. Ilgin, Ö. Yanartaş, Ç. I. Özgenç Biçer

**Affiliations:** ^1^Psychiatry; ^2^Neurology, Marmara University Research & Training Hospital, Istanbul, Türkiye

## Abstract

**Introduction:**

Aripiprazole is an atypical antipsychotic orally indicated for the treatment of schizophrenia, bipolar I, major depressive disorder. It is also indicated as an injection for agitation associated with schizophrenia or bipolar mania. Aripiprazole exerts its effects through agonism of dopaminergic and 5-HT1A receptors and antagonism of alpha-adrenergic and 5-HT2A receptors (Cosi et al., 2006). Ptosis is known as the drooping of the upper eyelid, and the patient usually presents with the complaint of the defect in vision and cosmesis (Shahzad & Siccardi, 2023). Orofacial dyskinesia and oromandibular dystonia are uncommon neurological disorders with involuntary, mainly choreic (dance-like) movements, or excessive, involuntary and sustained or repetitive muscle contractions that may involve the face, lips, tongue, and/or jaw (Bakke, 2016).

**Objectives:**

The aim of this study is to present a case of ptosis and orofacial spasm, which are neurological side effects that may be very rare side effects of aripiprazole.

**Methods:**

The 22-year-old woman was referred to the psychiatric service via the emergency service, due to thoughts of harming herself and irritability. The patient was planned to be hospitalized due to decreased sleep, increased speech, and persecution delusions for the last days. After the patient stayed in the service for 25 days, the patient was prescribed olanzapine 10 mg for discharge. At the follow-up appointment, it was learned that the patient had gained weight due to olanzapine and was switched to aripiprazole on 4th of July. The patient’s aripiprazole dose was gradually increased to 10 mg/day. The patient’s relatives gradually noticed drooping eyelids, involuntary oral movements, and impaired speech due to aripiprazole. Cranial MRI and cranial MRI angiography performed to rule out organic pathologies resulted normal. Accordingly, the aripiprazole dose was reduced to 5 mg/day by his mother, and at the last follow-up appointment on August 4, aripiprazole was stopped and paliperidone was started, and the neurological symptoms completely resolved 4 days later.

**Results:**

Since aripiprazole is frequently used in the field of psychiatry, its side effects are often wondered about. Extrapyramidal system side effects of antipsychotics are more common than ptosis. And In terms of the incidence of various extrapyramidal side effects, overall, no significant effects of age, sex, mean dose, study duration, or measuring method could be demonstrated. It is very important to distinguish between organic pathology and drug side effects, especially since ptosis accompanies neuromuscular diseases, ischemic, demyelinating brain lesions and intracranial aneurysms.

**Conclusions:**

As a result, patients who are started on antipsychotics should be closely watched for side effects to increase patient comfort and drug compliance. Also organic pathologies must be excluded before making a final decision that it is a drug side effect.

**Disclosure of Interest:**

None Declared

